# On the epistemology of resilience in public health: a novel perspective in a changing world

**DOI:** 10.3389/frhs.2024.1453006

**Published:** 2025-01-17

**Authors:** Alessandro Jatobá, Paula Castro-Nunes, Paulo Victor Rodrigues de Carvalho

**Affiliations:** Centro de Estudos Estratégicos da Fiocruz Antônio Ivo de Carvalho, Oswaldo Cruz Foundation (Fiocruz), Rio de Janeiro, Brazil

**Keywords:** health planning [MeSH], public health systems research (PHSSR), healthcare systems, health systems resilience, preparedness & response

## Abstract

This review proposes the foundations for an epistemology of resilience in public health, addressing the need for a theoretical framework to guide research and policy. Resilience, often ambiguously defined, is reexamined here as a critical attribute of public health systems, enabling them to adapt, absorb, and respond to routine and extraordinary demands without compromising universal and equitable service delivery. By integrating logical, sociological, historical, and philosophical perspectives, the paper delineates resilience as a structured and measurable concept, distinguishing it from common ambiguities in academic and policy discourse. It further introduces a set of foundational axioms to clarify the boundaries of resilience and support its operationalization within public health. These axioms emphasize the interplay between structural and functional dynamics, responses to internal and external stressors, and the importance of systems-level design over reliance on individual adaptations. This epistemological approach aims to bridge the gap between theory and practice, providing a robust basis for developing evidence-based policies that strengthen public health systems' ability to meet evolving challenges while promoting equity and universality.

## 1 Introduction

Challenges to the universal right to health and well-being—one of the Sustainable Development Goals listed by the United Nations (UN)—are numerous. Although population growth has slowed worldwide over the last decade, it remains rapid and is compounded by alarming poverty and inequality rates. The effects of climate change exacerbate these issues, particularly for vulnerable populations (1–4). Tensions between nations increase, leading to mass migrations, hunger, and diseases, as recent conflicts have demonstrated.

Recent health crises threaten to reverse the positive trends of mortality reduction that developing countries have accomplished over the past three decades, affecting the capacity of health systems to ensure broad access to essential services. In addition, new elites in low- and middle-income countries increased their demands for health services (5).

Several structural reforms implemented in the health sector in developing countries have positively affected access to healthcare, such as the More Doctors Program in Brazil and the expansion of health insurance in China. However, implementing sustainable reforms and maintaining long-lasting benefits are a challenge for policymakers.

Public health systems must be more adaptive, preventive, and responsive to guarantee universal, comprehensive, and equitable coverage of essential services amid dynamic and diverse demands (6). In this scenario, mobilizing resources to develop robust structures and institutional capacity has proven insufficient to overcome recent crises (7–9).

Thus, the discussion about the resilience of public and universal health systems is growing, even though there is still no clarity on what it means or consensus on how to operationalize it. In the wake of public health emergencies, properties such as preparedness, anticipation, responsiveness, or recovery have emerged in the literature, generating recommendations that do not explicitly mention resilience but may be confused with it, especially when dealing with the capacity of national health systems to respond to unusual occurrences.

However, the notion of resilience in the functioning of public and universal health systems, or what is considered a public health emergency, is diffuse. Most of the literature addressing resilience in public health still focuses on mobilizing acceptable responses to major public health events, such as epidemics and other health disasters, rather than the routine delivery of comprehensive health services (10).

In this article, a set of concepts is reviewed to establish an epistemology that defines reliable directions for research on the resilience of public and universal health systems. Pierre Bourdieu states that a scientific field is a structured space of positions whose properties can be independently analyzed (11). Laying the groundwork for an epistemology of resilience in public health will help delimit the boundaries of this field and consequently enable better structuring of scientific knowledge on this interdisciplinary topic around a shared theoretical framework. From logical and terminological perspectives, as well as political, sociological, philosophical, and historical viewpoints, this initiative serves as a cornerstone for an axiomatic definition of assumptions on this topic.

Epistemology often begins with a recognition of uncertainty surrounding a specific problem. The epistemology of resilience in public health concerns ensuring the universal right to continuous, high-quality, and responsive health services under variable circumstances in a changing world—a challenge that requires resilient performance from public health systems. Some foundational knowledge on this subject is self-evident, allowing the establishment of a set of initial empirical axioms.

Empiricism entails building knowledge that is grounded, testable, and open to revision—qualities essential for both intellectual rigor and practical application. Empiricism emphasizes real-world effectiveness, fostering technological advancements, scientific discoveries, and practical solutions. Moreover, the provisional nature of empirically derived knowledge allows for continual refinement as new evidence emerges, avoiding rigid, absolutist frameworks and enabling epistemologies to evolve. In this article, the proposed epistemology begins by establishing a foundational knowledge base, followed by a comprehensive articulation of the problem and four tentative axioms.

When developing an epistemology for resilience in public health, it is essential to align diverse concepts, principles, and perspectives. Such alignment ensures a meaningful contribution to the sustainable development of capacities needed to promote the universal right to health while adapting to fluctuating demands driven by inter-sectoral factors.

## 2 Resilience in socio-technical systems

The common understanding of resilience is the capacity of a system to anticipate, adapt, and reorganize under adverse conditions, and return to its original state of functioning (12–14). This definition is used in different disciplines, situations, and contexts that are not necessarily suitable for public health, in which adaptation cannot jeopardize service delivery, comprehensiveness, quality, or responsiveness. Moreover, returning to the initial state may not be acceptable, especially if the initial state is fragile (15).

Public and universal health systems have important structural and functional peculiarities (16–19). For instance, studies show that health systems continue operating during and after major public health events, unlike other complex systems, such as power plants or industrial facilities, that cease operations. By contrast, public health systems must absorb the impact of major events and continue to function; the recent conflicts in Ukraine and Palestine, for example, show that effective responses to complex, interrelated, and multidimensional events require methodological approaches that account for these complexities (20–22).

The use of the term “Resilience” in academia is still ambiguous, making it difficult to distinguish knowledge from opinions, hindering its methodical and reflective study, systematic development, and operationalization as a field for research, policymaking, and implementation. Studies on community resilience are hardly included in the canon of resilience due to heterogeneity in definitions and the lack of consistency on what is measured, an example of the effects of the ambiguous terminology (23, 24). Even where some cross-discipline consensus has been reached (e.g., the fields of disaster management and psychology have identified a similar set of factors associated with individual and collective resilience, such as the relationships and the need for a rapid post-disaster return to routine), there are still too few publications identifying key principles to guide evidence-based interventions in multiple systemic levels (from policy to care). Cutter et al. highlight linkages between resilience and vulnerability by refining a definition of resilience—as the ability to mitigate the damage caused by singular situations. These authors propose the Disaster Resilience of Place (DROP) model, for which resilience relies on sustainability, as previous conditions of the affected communities interact with the hazard event characteristics to produce immediate effects (25).

Bridging the epistemic gap between theory and practice in public and universal health systems resilience is essential to clarify the boundaries between scientific truth—that is, the critical evaluation of the validity of principles, hypotheses, and results of described knowledge—and mere belief. Developing an epistemology, distinguishing common sense and science, will clarify ideas and favor the approach of logical, semantic, and ontological problems related to resilience in public health.

Science develops in spaces in which ideas are confronted—often aggressively—by scientists with various points of view on a phenomenon, both disciplinary and practical, from different perspectives and assumptions of reality. In these spaces, epistemology makes it possible to structure a theoretical and practical body to a given concept, enabling its study from diverse perspectives and, thus, rational debate.

The field of public health needs to integrate different types of knowledge, usually from distinct epistemic communities and competing interests. When appropriately arranged, such articulation promotes the sharing of ideas and the development of more robust and efficient theories to support the formulation of evidence-based actions and policies.

Clear definitions of resilience turn it from an abstract ideal into a practical, measurable goal, allowing policymakers to create targeted policies that address immediate needs and strengthen systems for future disruptions. This leads to more robust, sustainable public health systems and societal stability. Haldane et al.'s study of COVID-19 responses in 28 countries, for example, highlights resilience-focused policymaking. The authors found that many nations enhanced resilience through governance by securing financing and reallocating resources, implementing support packages to stabilize businesses, protecting jobs, and assisting low-income populations. These measures helped people follow public health guidelines and reduced the burden on healthcare systems (26).

The following sections highlight problems, postulates, axioms, and implications for public health policies. The perspectives presented in this article are based on the extensive exploration of existing paradigms, history, and interdisciplinary relationships that bring existing knowledge on resilience closer to the field of public health.

## 3 Bridging health equity and universality with systemic resilience

Universality entails ensuring everyone has the right to access quality health services compatible with their needs, whenever and wherever they need them, from health promotion to prevention, treatment, rehabilitation, and palliative care, without access constraints (27, 28). To meet this challenge, countries need policies that encompass the needs of their populations and address the environmental and socioeconomic factors that affect health and well-being, including preparedness, responsiveness, and recovery from health emergencies. Coping with the complexity of providing universal and equitable care demands adaptive capacities from public health systems. These abilities enable systems to stretch finite resources to address the social determinants of health under diverse and often unpredictable circumstances.

Understanding the complexity of the social determinants of health is key to analyzing the epidemiological context of a given territory and identifying barriers to expanding access to services. Examples of such barriers include violence, misinformation, distrust, denialism, and other challenges that weaken health systems. Consequently, ensuring universal and equitable access to health requires fostering programs that continuously enhance systems' adaptive, absorptive, and preventive capacities, thereby strengthening their potential for resilient performance.

Several initiatives have demonstrated resilience in advancing universal and equitable health. These include special health programs for Indigenous Peoples and First Nations in countries like Brazil (29) and Canada (30), affirmative policies for minorities and marginalized populations (31), targeted interventions addressing emerging health challenges such as new diseases or teenage suicide, and the expansion of Community Health Worker programs in vulnerable areas in the United Kingdom (32).

Therefore, combining broad and uninterrupted coverage with access to good health services and acceptable social protection is essential. The concept of coverage is based on access, and the two ideas are therefore complementary. Without universal access, universal health coverage becomes an unattainable goal. To accomplish universal access, equitable right to health, and addressing social determinants, resilient abilities equip systems to adaptation and uninterrupted functioning of essential services. A resilient health system can not only withstand crises but also emerge stronger, more equitable, and better prepared to meet future challenges.

In the following subsections, we explore essential problems and the knowledge base, providing axioms and a starting point for debate on the methods and theories of resilience in public health and how the idea contributes to meeting the challenge of the universal and equitable right to health in the 21st century.

### 3.1 Problem

In the second half of the 18th century, urbanization was prioritized, especially in large European cities, to organize residents cohesively and homogeneously. The mobilization of resources to improve the quality of life of the population had its principal motivation in economic aspects, given the value of the workforce.

In addition, the struggle between the rich and the poor, plebeians and bourgeoisie, created the need for political health authority. The bourgeoisie adopted a model based on intervention, using surveillance and hospitalization, evolving from the political-medical quarantine schemes of the late Middle Ages. Public hygiene, derived from these quarantine measures, boosted the development of urban medicine from the second half of the 18th century onwards. In a global context, life expectancy at birth has increased from 47 years in the mid-20th century to around 70 years today and is projected to reach 76 years by the mid-21st century (33).

In 1920, the Dawson report included a critique of the Flexnerian model based on a perspective of rationalizing the delivery of health care to meet specific social demands. It proposed restructuring the model of care in services organized according to the levels of complexity and costs of treatment, similar to modern healthcare networks (34).

Foucault defines this period as the birth of social medicine, highlighting a strategy of biopower, that is, social domination over individuals through regulating their lives (35). This included public health campaigns to regulate behaviors and lifestyles, medical procedures, and interventions in educational systems. Another major object of urban medicine, according to Foucault, is the organization and control of water and sewage sources, since urban disorder and the lack of basic sanitation were considered responsible for the main epidemic diseases in the second half of the 14th century.

Although aspects such as disease prevention, continuous well-being, and health promotion originate in much older societies, the delimitation of a widely accepted concept of public health became accepted from the Industrial Revolution onwards, culminating in the publication of the Charles-Edward Winslow manifesto in 1920 (36).

Winslow emphasizes the comprehensive, collaborative, and interdisciplinary nature of service provision focused on maintaining people's health and well-being. This definition is pertinent and highlights the complexity of developing comprehensive public health systems.

Regarding the systematization of care, historically unstable service delivery demands adaptation at different levels. Resources are always limited and demands have their own dynamics, so health systems, governments, service providers, and citizens must adapt to constant change (37). It is natural that the outcomes of collaborative health production will vary, with positive and negative results.

That is why strengthening the intrinsic adaptive capacity of health systems and its resilience involves responding to both routine and extraordinary demands. Often this ability to adapt is relegated to the behavior of people—a kind of informal resilience—that is not under the control and knowledge of the governance layer. For example, when community health workers in violent locations adapt their house call schedules to avoid the times when confrontations between the police and local criminals occur, they are, indeed, exhibiting resilience (38). Managers and policymakers should, however, analyze such variability and design tailored policies to support adaptations or mitigate the negative effects of the context to prevent disruption—like possible harm to community health workers during house calls. To support resilience, making it part of the health management process, it is necessary to adequately identify the effects of this adaptive capacity on the variability of outcomes of essential public health functions.

The literature on recent public health events, such as the COVID-19 pandemic and the 2024 dengue outbreak in Brazil, confirms that public health systems adapt to continue functioning during unintended events. The post-COVID literature is replete with innovative practices, adaptations, and actions carried out during the pandemic (18, 26, 39, 40). However, these initiatives are usually followed by an interruption to regular procedures, such as elective surgery or the management of chronic patients, which can lead to pent-up demand, increased queues, and a worsening of a population's health conditions. It is evident that once hit by public health events of national or international importance, system capacity is stretched, but routine operations must remain responsive because regular demands never pause.

It is no coincidence that during the recent dengue outbreak in Brazil, the number of COVID-19 cases unexpectedly increased. Because the dengue outbreak hit during Carnival festivities, the Brazilian Unified Health System (SUS) had to handle an unexpectedly complex epidemiological scenario with limited resources, especially in densely populated locations.

To cope with emerging diseases, it is necessary to understand their specifics and peculiarities, in addition to understanding social determinants and conditioning factors of health. Confronting COVID-19 required robust institutional capacity, such as intensive care beds, ventilators, and skilled professionals, which are not necessarily the same resources for other outbreaks. Dengue is an arbovirus with characteristics related to territory and rainfall density, as well as the environmental evolution of the vector, which reproduced in clean water and now reproduces in dirty water. Adapting to these dynamics is far from trivial.

The epidemiological situation in resource-limited settings, such as in low- and middle-income countries, implies a triple disease burden, differing from the classic Omramian (41) epidemiological transition. This triple burden involves, simultaneously, an agenda of communicable diseases, malnutrition, and reproductive health issues; chronic non-communicable diseases and their risk factors (such as smoking, overweight, obesity, sedentary lifestyle, stress, and rich ultra-processed diets); and an increase in external causes (accidents), as the primary causes of morbidity and mortality.

### 3.2 Fundamentals

Described with slight variations in the publications on the subject, Health Systems Resilience is defined by the World Health Organization (WHO) as the capacity of all health-related actors and functions to collectively prepare responses, mitigate and recover from disruptive events with implications for public health, maintaining the provision of essential functions and services and of using past experiences to adapt and positively transform the health system (42).

According to the WHO, the key attributes of a resilient health system include awareness of its capabilities and risks; mobilization and coordination of resources for effective risk management; self-regulation for threat response through evidence-based decision-making; adaptation, as needed, to withstand harsh conditions; comprehensive and good quality provision of the necessary services in all contexts; and identifying and using lessons learned to improve and transform, while ensuring integration between health security, systems strengthening, and other health programs.

The European Union Working Group on Health Systems Performance Assessment also defines resilience to cover the main characteristics described in the literature from different areas, from Engineering to Social Sciences. For this group, resilience describes the ability to proactively predict, absorb, and adapt to shocks, and make structural changes in a way that allows systems to sustain necessary operations, resume optimal performance as quickly as possible, and transform their structure and functions to strengthen the system and reduce its vulnerability to future similar shocks.

Despite the respectability of both organizations and the convergences between their definitions of resilience, there are still gaps to fill. The literature on complex systems management does not offer a consensual definition of organizational resilience. Even less is said about resilience as an attribute to be developed in organizations dealing with unstable and adaptive contexts such as public and universal health systems.

In the most well-known definitions, including the two mentioned above, convergence on the idea of adapting to disruptive shocks or unexpected major events is noteworthy, although not likely, given the dynamics of the implementation of public policies and the consequent provision of comprehensive and responsive health services that require continuous development of resilience. Even when responding to major public health events, the key to resilient performance lies in the characteristics developed under normal conditions (19, 26, 43). For instance, continuous monitoring of long-term threats makes it possible to anticipate shocks and respond resiliently to extreme situations, as demonstrated in Arcuri et al.'s study (43). The authors show how continuous mapping of constraints in service delivery to remote fluvial regions reinforced the importance of regular protocols for health service provision, which could be mobilized effectively during major crises.

Similarly, returning to a pre-shock state makes no sense if service delivery was previously fragile, incomprehensible, or non-responsive. Resilience in public health, therefore, involves permanent transformation based on continuous learning (44).

In other situations, resilience has been described as the organizational capacity to deal directly or indirectly with conditions faced by populations and to recover from unexpected disturbances (18, 45). However, this definition is still limited because it does not include various aspects of complex systems, such as those relating to public health. In addition to the quantity and frequency of disorders that need to be processed and absorbed to ensure the quality of routine assistance, their essential functions cannot be interrupted. Examples of essential functions halted amidst COVID-19 were elective surgeries in Brazil, as some of them had to be postponed, straining already overloaded healthcare queues (10, 46).

It is worth noting, however, that despite the visible—and expected—gaps, there are plenty of publications of experiences with chronic structural long-term stresses. It is important, however, to go beyond the ability to withstand the effect of external shocks on the delivery of health services. From this perspective, the probability of interrupting routine services during an unexpected event is explicitly recognized; a system's capacity to mitigate impact, take corrective measures, readjust its operation to the new context, and learn from experience is conceived as more than the sum of the available financial, material, and human resources, that is, the system's institutional capacity (8).

This delimitation is an important step in fitting public health resilience into a framework, understanding that the boundaries of the definitions might not be shared. The clear distinction between sudden shocks, slower impacts, less intense events, and chronic stressors helps establish the view that health systems must be constantly resilient. The ability to dampen changing conditions and unexpected development poses new challenges—or opportunities—to provide quality services in the face of ongoing variability, such as organizational and political instability, workforce turnover, patient expectations, and so on.

### 3.3 Axioms

To cope with fluctuating demand, dynamic contexts, and resource constraints, public and universal health systems depend on the continuous articulation of multiple and flexible interdependent processes. Adaptation is an intrinsic element, but it needs to be developed sustainably.

The contrast between successful and unsuccessful cases of sustainable adaptation has been examined in the context of socio-technical systems design (47); in most studies, the concept of brittleness refers to the pressure on operational limits. Health systems are always being stretched to accommodate challenging situations; when pushed too far, they become more prone to such a condition. Usually, the strategy for these situations is temporary, such as implementing solutions to problems whose consequences have already materialized. We observe this when field hospitals or exceptional vaccination programs are carried out to tackle the spread of outbreaks.

Although the importance of such actions cannot be underestimated, they must generate knowledge enabling permanent adjustments, not mere performance compensations (48). Adaptation is the cornerstone of axioms of resilience in public health, as is explained in the following subsections.

Many frameworks have addressed resilience in complex systems, including public health, though most focus on disaster response. The WHO's Building Blocks framework merits particular attention (26, 45). It is also important to note that, in general, they overlook variability and adaptive capacity at more abstract levels than is necessary to implement public policy.

The experience with recent propositions on strengthening resilience, more or less specifically applied to public health, entailed a set of axioms that start from the idea that resilience in public health resides in the ability of national health systems to manage volatility in the outcomes of essential public health functions, to make it possible to sustain uninterrupted and resolutive functioning of routine services at the same time the systems adapt to eventual demand fluctuations beyond its usual capacities (49, 50).

Axioms result from generalizations based on empirical observation. In social sciences, especially where there's not a dominant paradigm yet (51), the establishment and validity of axioms result preliminary from studies with similar results that corroborate their premises. Thus, as in the rationalist tradition, axioms originate from self-evident and truthful premises and regulate postulates, supporting demonstrations of other empirical conclusions.

Axiom 1 focuses on the distinction between the strengthening of structures and the functioning, and how this interferes with the capacity of public health systems; Axiom 2 discusses the influence of internal and external events on the adaptive capacity of health systems; Axiom 3 is dedicated to the need to deal with large and eventual occurrences, as well as small and routine events, for resilient performance; Finally, Axiom 4 indicates that adaptation cannot rely exclusively on workers' initiatives, but arise from formal systems design.

An epistemology must accompany a set of premises gathered in various empirical studies making up the field's knowledge base, rationalizing a line of thought on a topic and bringing to light philosophical, semantic, and gnoseological assumptions, methods, or results of contemporary scientific investigations (52, 53). Table 1 presents noteworthy resilience frameworks that address or apply to public health.

**Table 1:** Selected resilience frameworks.

Framework	Principles	Axiom 1		Axiom 2		Axiom 3		Axiom 4	
		Structure	Functioning	Exogenous events	Endogenous events	Major events	Minor events	Workers	Systems design
The building blocks of climate resilient health systems (54)	Each building block relates to a set of structural attributes of health systems, which, in theory, can be measured and evaluated, sometimes quantitatively, sometimes qualitatively.	X				X			X
Health Emergency and Disaster Risk Management Framework (55)	Functions are organized thus: policies, strategies, and legislation; planning and coordination; human resources, financial resources, information, and knowledge management: risk communications; health infrastructure and logistics; health and related services; community capacity for health; monitoring and evaluation	X		X		X			X
Kruk et al. (39)	The resilience of a health system is the ability of health actors, institutions, and the population to maintain their essential functions when adversity arises and to reorganize based on the lessons learned during a crisis	X	X			X		X	X
Hyogo Framework for Action 2005–2015 (56)	Building resilient communities to reduce personal, social, economic, and environmental loss	X		X		X			X
Cutter et al. (25)	Community resilience based on educational equality, income, social capital, health access, mitigation plans, religious affiliations, community aspirations, emergency management assets, mitigation activities, infrastructure, and buildings	X							
Ungar (14)	Seven principles for resilience in psychosocial and cultural systems: Resilience occurs in contexts of adversity; Resilience is a process; There are trade-offs between systems when a system experiences resilience; a resilient system is open, dynamic, and complex; a resilient system promotes connectivity; a resilient system demonstrates experimentation and learning; a resilient system includes diversity, redundancy, and participation		X		X	X			X

#### 3.3.1 Axiom 1: demand fluctuations require synergy between structure and functioning

Health system structures encompass resources, beds, workforce, funding, and other attributes that build on institutional capacity. Functioning concerns how such structure operates, such as governance arrangements, protocols, and operational procedures.

Acknowledging resilience as a capacity—or a set of capacities—of the health system does not prevent considering the circumstances in which systems operate and allow (or not) the emergence of resilient attributes. Based on attributes and structures, it is necessary to understand the skills and capacities public and universal health systems use to absorb, react, adapt, or transform when dealing with different types of events, guided by implicit and explicit rules, and by the variety of decisions and interactions of actors—patients, technicians, managers, politicians, and private companies.

These interactions depend on people's mindsets and interests, as well as on physical structures within the context in which different services operate. Managers, workers, patients, and their communities are the basis for the functioning of resilient health systems. However, governance and political arrangements need to enable spaces and competencies for adaptation to improve system performance.

Resilience in public health needs to accommodate both structures and functioning. It is not possible to conceive or assess resilience based on structures alone, as recent experience has shown (57). On the other hand, essential functions of health systems, no matter how well-designed, require adequate and strong structures, whether for routine operations or responses to major public health events. The challenge lies in promoting resilient health systems capable of optimal functioning in routine and unforeseen situations.

The response to COVID-19 highlights how inadequate combinations of structure and functioning can exacerbate systemic fragilities. Carvalho et al. (15) illustrate this through the case of Brazil, where political arrangements hindered the timely acquisition of vaccines despite a substantial budget being available. Additionally, anti-vaccine rhetoric and political pressure on local authorities caused significant delays in vaccine distribution operations, even though resources had been allocated appropriately.

#### 3.3.2 Axiom 2: resilience is about coping with events of different magnitudes

This axiom stems from a recurring question: Is the resilience needed to function under crises (pandemics, natural disasters, major accidents, and technological innovation) the same as under routine operations?

It is important to emphasize that resilience and brittleness are not contingent upon acute events. Numerous events of different intensities occur daily, and resilience lies in developing attributes that keep systems prepared for an event of any nature or intensity, especially in the intrinsically unstable context that involves the delivery of public health services.

Resilient attributes are activated whenever necessary, on both routine and exceptional provision of care. Stability and the development of shock-absorbing capacity are very present themes in disaster management. Thus, the concepts outlined in this field are widely known. However, developing resilient behavior involves mobilizing efforts to anticipate future failures, in addition to prompt responses.

Agents in a system must find innovative ways to perform in new situations. As their situational awareness changes, procedures must change, allowing the mobilization of critical resources and creative action that support responsiveness. Regarding the resilience of public health systems, this communication also relates to the capacity for collaboration with civil society, as community engagement strengthens the skills that facilitate resilience (58).

Disease outbreaks are not the only risks to people's health. Although there are very sudden events, there are also those factors that develop over long periods, such as the droughts and floods in northeastern and southern/southeastern Brazil, respectively. Small-scale events with limited consequences occur regularly, while others can lead to catastrophic consequences for public health.

#### 3.3.3 Axiom 3: public health functions are influenced by both endogenous and exogenous variables

A question little addressed in the literature on resilience of complex systems is whether the adaptive processes for external and internal events are the same. Frameworks focused on resilience to major events such as natural disasters, epidemics, or climate change propose that system structures are mobilized to deal with exogenous variables (54, 56). Even the frameworks dedicated to the functioning of systems are still focused on adverse events of exogenous nature.

In systems theory, resilience is a process or set of processes needed to handle internal and external threats, variables, or situations, rather than a static or structural characteristic of systems (13, 14, 59). Processes that contribute to more sustainable systems in contexts of adversity include (a) persistence; (b) resistance; (c) recovery; (d) adaptation; and (e) transformation (60).

Persistence allows systems to function internally and externally, even when stressed. Despite stability makes systems look like they're idle or balanced, persistence usually takes effort and resources. While persistence enables systems to continue performing with the necessary support, resistance indicates the risk of overload by internal or external stressors that exhaust resources. Resistance allows a system to continue functioning even when a disturbance is present. Examples include communities that rebuild residences in areas at risk when public policies do not provide acceptable housing locations.

The recovery process is conceptually problematic for public and universal health systems, as it implies a return to previous functioning states that may no longer be appropriate. Moreover, returning to the previous state is unlikely if new information and functionality have been introduced to cope with the disruption.

Adaptation is a diffuse set of multi-level and multi-scale simultaneous interactions. While recovery returns a system to an earlier state, adaptation means adjusting and learning to operate during disturbances. The system changes its operation to be sustainable or to maintain stability, facilitated by simultaneous changes that accommodate this new focus.

Transformation emerges when a system radically turns into something new, e.g., a change in primary care policy with the introduction of community health workers (32). Transformation, however, like adaptation, describes the outcomes of changes but does not predict the timing or desirability of such changes.

#### 3.3.4 Axiom 4: resilience must be achieved through systems-level design and not solely through individual adaptations

Systems that depend only on people's resilience overload workers and increase the possibility of resonating variations and instability. Informal resilience, which emerges from unobserved adaptations, can lead to a loss of control (61). Organizations should foster management processes, technologies, and cultural shifts focused on resilience skills.

For Hollnagel (62), resilient performance emerges from the ability to respond, monitor, learn, and anticipate. These abilities are called *systemic potentials for resilient performance*:

• Responding to events: how to respond to regular, novel, and disruptive events.
• Monitoring what is happening: what already is or may become a threat in the short, medium, and long term. Monitoring should encompass what occurs in the context in which the health organization operates. It involves the ability to oversee what is critical.
• Anticipating future threats and opportunities: assessing potential disruptions, pressures, and consequences, that is, addressing problems that have not yet manifested.
• Learning from past failures and successes: learning from experience and learning the right lessons from the right experiences.

The WHO also highlights skills centered on the functioning of essential public health functions and the broader determinants of health, equity, and resilience (42). These skills—awareness, mobilization, diversity, self-regulation, integration, adaptability, and transformation—form a conceptual framework that overlaps with Hollnagel (62), underscoring the importance of a systemic-level design that supports people's adaptive abilities.

Also with some overlap, the World Bank lists resilient skills. In that case, resilient health systems are aware of threats; agile in responding; shock-absorptive; adaptive; and able to transform permanently based on lessons learned (63). This idea highlights the importance of inter-sectoral articulation, involving government and society and combining preparedness and provision of services, political actions, and investments in public health.

## 4 Implications for public policies

Resilience in public health is a contextual property dependent on past pathways, so building resilient public and universal health systems requires ongoing efforts to strengthen governance and effective inter-sectoral partnerships (64). In such systemic logic, resilience is an essential feature of organizational management, the promotion of collective skills, and responses to structural and political pressure (65, 66). Therefore, the resilience of health systems must be intrinsically linked to public health policies, through a systemic approach involving organizations and society. The challenges encompass both basic and implementation sciences (67).

First, existing resilience frameworks imply both positive and negative outcomes. The focus is not only on adaptive failures (traditionally highlighted in accident analyses) but also—and more importantly—on the adaptations and corresponding circumstances that led to known outcomes. From an intervention perspective on public health constraints, therefore, implementing policies for resilience implies encompassing preventive actions (at the primary care level) rather than simply trying to recover from disruptions.

Secondly, even in cases where disruption is inevitable, the resilience approach implies an emphasis on addressing known deficiencies and continuously improving aspects that are already strong. An example of resilience in the face of inevitable disruption can be found in the approach of the SUS during the extreme floods in Rio Grande do Sul in 2024. Despite extensive damage to infrastructure and service interruptions, the SUS was able to promptly activate the National Force (FN-SUS), a specialized task force designed to respond to emergencies and disasters that affect the health conditions of people (68).

When the storm hit, the FN-SUS articulated multiple system—involving the police, firefighters, and civil defense rescuers—to maintain essential operations and prioritize the safety of patients. While disruptions to energy systems and patient transport were unavoidable, the focus on shoring up known vulnerabilities in critical infrastructure and refining evacuation protocols allowed for a more robust response. This experience also informed continuous improvements, leading hospitals to enhance flood-proofing and emergency preparedness for future extreme weather events.

The third critical feature is that the effective implementation of resilience in public health involves a commitment to understanding the underlying processes of vulnerability and the protective factors of populations. Identifying functions that show significant links between context and the results of adjustments to manage variability is only the initial step; the goal is to clarify whether the various potential mechanisms for implementing resilience are implicated or available to address the effects of vulnerability so that proper guidelines, methods, and practices for intervention or implementation can be carried out.

In sum, resilience encompasses a scientific approach with multiple characteristics. Resilience in public policy demands attention to (a) the positive results of adaptations in the presence of adversities at any level; (b) evidence-based knowledge about vulnerability and protection mechanisms that may be unique; (c) shifting the focus from correcting failures or mismatches to considering competence in successful adaptations (implicitly emphasizing prevention); (d) attention to both the problems and strengths of individuals in vulnerable populations (as both make interventions possible); and (e) the systematic exploration of processes explaining or substantiating links involving vulnerability and empirically identified protective factors.

Healthcare models are sociotechnical combinations structured to solve prevalent problems and meet the overall needs of populations, whether individually or collectively. These models result from the intermediation between technical and political elements. Therefore, promotion and prevention should not be neglected in favor of emergency actions, which often rely on campanist strategies, focusing primarily on controlling endemic diseases and outbreaks from individual or hospital-centered perspectives (fragmented, Flexnerian, and medically oriented). Resilient public and universal health systems must prioritize continuity in public health, not just during moments of crisis. Directives like Universality and Equity, while demanding resilient performance, also facilitate the emergence of conditions for resilience within the system.

Finally, validating a new epistemology depends on confronting its initial assumptions with emerging empirical evidence. The scientific debate, alongside practical applications, serves as an arena for refining the iterative process of questioning, testing, and revising knowledge and ensuring that an epistemology remains relevant, adaptable, and effective. This process of validation requires a continuous dialogue between theory and evidence, where assumptions are regularly scrutinized, and frameworks are adjusted based on new findings. It is through this dynamic and reflective process that the epistemology of resilience in public health will gain its strength, credibility, and applicability.

## Data Availability

The original contributions presented in the study are included in the article/Supplementary Material, further inquiries can be directed to the corresponding author.
